# Quantifying the Impact of Different Dietary Rumen Modulating Strategies on Enteric Methane Emission and Productivity in Ruminant Livestock: A Meta-Analysis

**DOI:** 10.3390/ani14050763

**Published:** 2024-02-29

**Authors:** Bulelani N. Pepeta, Abubeker Hassen, Eyob H. Tesfamariam

**Affiliations:** 1 Department of Animal Science, University of Pretoria, Private Bag X20, Hatfield, Pretoria 0028, South Africa; nangamso.pepeta@gmail.com; 2 Department of Plant and Soil Science, University of Pretoria, Private Bag X20, Hatfield, Pretoria 0028, South Africa; eyob.tesfamariam@up.ac.za

**Keywords:** methane emission, mitigation, production performance, ruminant livestock

## Abstract

**Simple Summary:**

Consumer perception related to health and environmental issues associated with ruminant products, such as greenhouse gas emissions, has led to a paradigm shift aimed at mitigating the potential harmful effects of ruminant production. This study consolidated the current body of research on dietary rumen manipulating strategies with an aim to quantify their impact on rumen fermentation, enteric methane (CH_4_) emission and productivity by creating a global database on in vivo evaluation studies. A meta-analytical approach was used to achieve the study’s aim and the result showed that nitrate, saponin, oils, biochar and 3-nitroxypropanol (3-NOP) were effective dietary rumen modulating strategies to mitigate enteric CH_4_ emission. Of these effective strategies, oils and 3-NOP provided a co-benefit in terms of improving productivity in ruminant livestock. Concentrate feeding equally improved production without any significant effect on enteric methane emissions.

**Abstract:**

A meta-analysis was conducted with an aim to quantify the beneficial effects of nine different dietary rumen modulating strategies which includes: the use of plant-based bioactive compounds (saponin, tannins, oils, and ether extract), feed additives (nitrate, biochar, seaweed, and 3-nitroxy propanol), and diet manipulation (concentrate feeding) on rumen fermentation, enteric methane (CH_4_) production (g/day), CH_4_ yield (g/kg dry matter intake) and CH_4_ emission intensity (g/kg meat or milk), and production performance parameters (the average daily gain, milk yield and milk quality) of ruminant livestock. The dataset was constructed by compiling global data from 110 refereed publications on in vivo studies conducted in ruminants from 2005 to 2023 and anlayzed using a meta-analytical approach.. Of these dietary rumen manipulation strategies, saponin and biochar reduced CH_4_ production on average by 21%. Equally, CH_4_ yield was reduced by 15% on average in response to nitrate, oils, and 3-nitroxy propanol (3-NOP). In dairy ruminants, nitrate, oils, and 3-NOP reduced the intensity of CH_4_ emission (CH_4_ in g/kg milk) on average by 28.7%. Tannins and 3-NOP increased on average ruminal propionate and butyrate while reducing the acetate:propionate (A:P) ratio by 12%, 13.5% and 13%, respectively. Oils increased propionate by 2% while reducing butyrate and the A:P ratio by 2.9% and 3.8%, respectively. Use of 3-NOP increased the production of milk fat (g/kg DMI) by 15% whereas oils improved the yield of milk fat and protein (kg/d) by 16% and 20%, respectively. On the other hand, concentrate feeding improved dry matter intake and milk yield (g/kg DMI) by 23.4% and 19%, respectively. However, feed efficiency was not affected by any of the dietary rumen modulating strategies. Generally, the use of nitrate, saponin, oils, biochar and 3-NOP were effective as CH_4_ mitigating strategies, and specifically oils and 3-NOP provided a co-benefit of improving production parameters in ruminant livestock. Equally concentrate feeding improved production parameters in ruminant livestock without any significant effect on enteric methane emission. Therefore, it is advisable to refine further these strategies through life cycle assessment or modelling approaches to accurately capture their influence on farm-scale production, profitability and net greenhouse gas emissions. The adoption of the most viable, region-specific strategies should be based on factors such as the availability and cost of the strategy in the region, the specific goals to be achieved, and the cost–benefit ratio associated with implementing these strategies in ruminant livestock production systems.

## 1. Introduction

Livestock contribute to approximately 14.5% of global agricultural-related greenhouse gas (GHG) emissions [1], with ruminants being responsible for about 80% of these emissions [2]. Enteric methane (CH_4_) emitted by ruminants from ruminal fermentation accounts for 2–12% of the energy loss from the diets they consume [3]. In order to improve the energy utilisation efficiency of ruminant diets and reduce their CH_4_ emissions, it is necessary to adopt sustainable dietary strategies that are specifically designed to mitigate CH_4_ emissions. Several dietary strategies to reduce CH_4_ emissions in ruminants have been extensively studied and qualitatively reviewed [4,5]. However, the results of these studies have been variable. Therefore, a meta-analytical approach could provide quantified effects of these strategies on enteric CH_4_ emissions and productivity in ruminants, improving overall understanding. Most of the available meta-analytical studies have only focused on individual strategies at a time [6,7], whereas studies evaluating multiple rumen-modulating strategies were based on observations from a sole ruminant species [8]. However, van Gastelen et al. [9] quantitatively reviewed the effects of several enteric CH_4_ mitigating strategies on how they behave across different ruminant species and conclusively reported no difference between species when the mode of action directly approaches a methanogenesis pathway. Hence, there is a need to complement this approach by consolidating data from global studies conducted to evaluate different dietary rumen manipulating strategies in ruminants using percentage change data rather than using absolute values, as the approach by previous authors ruled out animal species as an explanatory variable when quantifying effects of methane reducing strategies in relation to the biological influence of methane-related pathways. Moreover, Congio et al. [10] compared different rumen-modulating strategies for ruminant livestock, but their study was limited to the Latin America and Caribbean regions. Thus, there is a need to conduct a quantitative review at a global level in order to identify the dietary rumen modulating strategies that reduce the enteric methane emission and/or those that increase productivity across ruminant livestock systems. Recently, Arndt et al. [11] examined the quantified effects of sizeable dietary CH_4_ mitigating strategies without accounting for rumen fermentation parameters or the quality of milk produced in response to these strategies. Such limitations hinder the translation of results from these studies due to the need to determine the feasibility of effective CH_4_ mitigation strategies and their impact on rumen fermentation, feed efficiency and production parameters like milk yield and milk quality. Therefore, the current meta-analysis study was conducted using global in vivo experiments to quantify different dietary rumen modulating strategies such as the use of plant-based bioactive compounds (saponin, tannins, oils, and ether extract), feed additives (nitrate, biochar, seaweed, and 3-nitroxy propanol), and diet manipulation (concentrate feedings) on enteric methane emissions (CH_4_ production, CH_4_ yield and CH_4_ intensity) and the productivity of ruminant livestock.

## 2. Materials and Methods

### 2.1. Dataset

**Figure 1:**
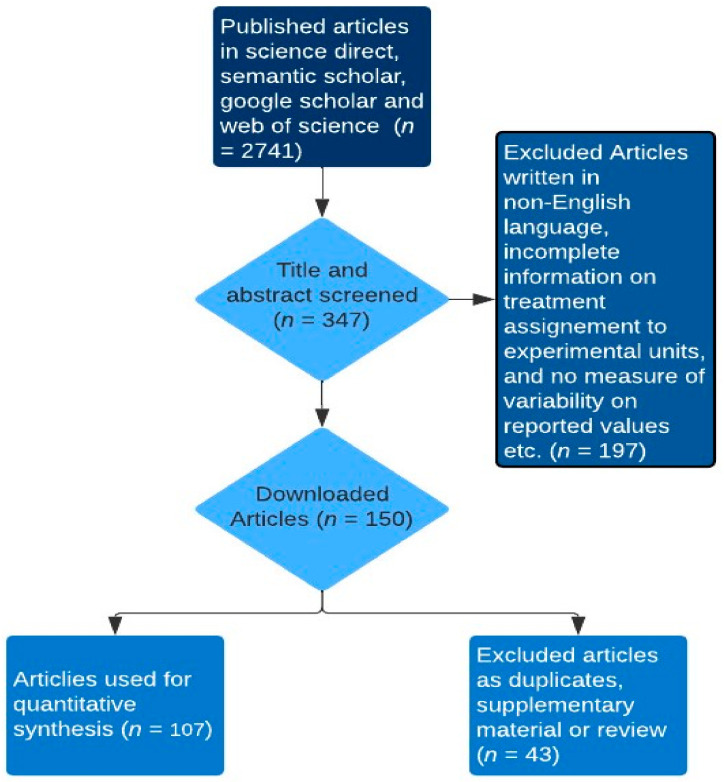
Flowchart of article selection.

To comparatively examine quantified effects of multiple rumen-modulating strategies on enteric methane (CH_4_) emission metrics such as CH_4_ production (g/day), CH_4_ yield (g/kg dry matter intake) and CH_4_ intensity (g/kg meat or milk), dry matter intake, total tract digestibility, ruminal fermentation parameters, milk yield, liveweight gain, and feed efficiency of ruminants, we firstly identified strategies with modes of action associated with methane formation-related pathways. A key rationale behind this approach stems from the notion that if the mode of action of a dietary methane mitigation strategy is closely tied to methanogenesis-related fermentation pathways, it is likely to yield consistent outcomes across diverse ruminant types [9]. Thereafter, the dataset was created by searching online internet sources (i.e., google scholar, web of science, semantic scholar and science direct) for peer-reviewed articles, conferences and theses using the search terms: in vivo, rumen modulating strategies (tannin, saponin, ether extract, nitrate, concentrate feeding, oils, biochar, seaweed, and 3-nitroxy propanol), ruminants (cattle: dairy or beef, buffaloes, goats, sheep, and deer), methane production or emission, and production performance (dry matter intake, total tract digestibility, average daily gain, milk production and yield, and feed conversion ratio) in different combinations. It is important to note that the data on average daily gain and feed conversion ratio (an index of feed efficiency) were gathered from studies reporting the growth performance of beef cattle, buffaloes, deer, sheep, and goats, excluding dairy ruminants. Similarly, feed efficiency in dairy ruminants, measured in grams of milk produced per kilogram of dry matter intake (g/kg DMI), was derived from studies specifically conducted to evaluate milk production data in dairy ruminants. Additionally, ether extract (EE) is the crude fat containing other compounds extracted by diethyl or petroleum ether prior to hydrolysis. The addition of EE as an explanatory variable in mathematical models that predict CH_4_ emission from ruminants improved the accuracy of the predictions [12]. Hence, EE was evaluated as a separate strategy from the oil addition strategy in this current study. The titles and abstracts of the publications were used as the first screening approach to identify the suitability of the publications for inclusion in the database. Studies were further assessed if they reported experiments conducted using ruminants, evaluated the CH_4_ mitigating strategies in question, and whether the treatments within each study were clearly defined for inclusion in the database. Publications included in the final dataset as depicted in, met the following criteria: (1) studies should have been written in English; (2) studies should have reported in vivo experiments; (3) methane emissions data reported were measured (e.g., sf_6_: sulphur hexafluoride) and not estimated; (4) production performance data were reported; (5) for any variable of interest, studies had to have reported the mean and the measure of variability such as the standard error of the mean or the reported measure of variability allows the calculation of the standard error of the mean; (6) studies should have reported the chemical composition per treatment (Figure 1). The dataset included 107 studies published from 2005 to 2023, conducted in 27 countries across six continents viz. Africa, Asia, Australia, Europe, North America, and South America. Studies including more than one methane mitigating strategy as treatments (e.g., factorial experiments) were counted as many times as the variation shown for rumen modulating strategy classification criteria. For instance, studies that compared different strategies and their combinations (interaction) were counted twice, and the data from the interaction effects were not included in the dataset. Sources of saponins encompassed tea saponin, *Linum usitatissimum*, *Medicago sativa*, *Garcinia mongostana* (peel powder), *Macrotyloma uniflorum*, *Camellia sinensis*, *Gliricidia sepium*, *Enterolobium cyclocarpum*, *Pyrus salicifolia*, *Yucca schidigera*, and *Quillaja saponaria*. Tannin sources included *Varchelia tortilis*, *Terminalia chebula*, *Allium sativa*, *Azodirahta indica*, *Artcarpus heterophyllus*, *Ficus benghalens*, *Corylus avellana*, *Kobe lespedeza*, *Garcinia mangostana*, *Cymbopogon citratus*, *Matricaria chamomilla*, *Sercea lespedeza*, *Cosmos bipinnatus*, hydrolysable tannin extract, *Camellia sinensis*, condensed tannin extract, *Gliricidia sepium*, *Enterolobium cyclocarpum*, and *Schinopsis lorentzii*. Oils were derived from *Zea mays*, *Allium sativa*, *Helianthus annus*, *Linum usitatissimum*, *Brassica napus*, *Menthae piperitae aetheroleumm* and *Trachyspermum ammi*. Ether extract was from crude and extruded *Linum usitatissimum*, *Oryza sativa* straw, *Garcinia mangostana* peel, *Camellia sinensis*, *Digitaria eriantha*, *Punica granatum*, *Madhuca longifolia*, *Tecomella undulata*, *Elaeis oleifera* meal, and *Cuminum cyminum*. Biochar sources included *Pinus Sylvetris*, *Gallus gallus domesticus*, (droppings), *Bambusa vulgaris*, *Oryza sativa* (husks), *Pistachia vera* (by-product), *Junglus* (shell). Seaweed was derived from *Ascophyllum nodosum*, *Asparagopsis taxiformis*, *Sargassum wightii*, and Rhodopyta.

### 2.2. Calculations and Statistical Analysis

The level of the response variables varied greatly across studies using different types of ruminants, making comparisons on absolute values across studies impractical. Comparison using the mean effect size approach promoted feasibility and facilitated the examination of dietary rumen modulating strategies using data from different types of ruminants. The influence (mean effect size, %) of the dietary rumen modulating strategies on any response variable was calculated as the difference between the rumen modulating strategy (mitigating treatment) and the respective control treatment or baseline, divided by the control treatment, and multiplied by hundred (100) for percentage representation [13], as follows:

(1) $$\frac{mitigating\;treatment-control\;treatment}{control\;treatment}\times 100$$

The dataset was subjected to the mixed model procedures of SAS (9.4 SAS Inc., Cary, NC, USA) and different studies were considered as random effects. The initial body weight of animals and day of lactation in dairy ruminants were considered as covariates. The dietary rumen modulating strategies, namely saponin, essential oils, tannins, nitrate, concentrate feeding and ether extract, seaweed, and 3-nitroxypropanol, were considered as fixed effects. To account for the differences arising from studies used in the current evaluation, the inverse of the squared standard error of the mean was used as the weighting factor in the “weight” statement of the model as outlined by St-Pierre [14]. The statistical significance was declared at *p* < 0.05.

## 3. Results

Table 1 presents summary statistics of the refined complete dataset on dietary rumen modulating strategies, initial body weight, dietary composition, intake, digestibility, average daily gain, milk parameters, methane and fermentation parameters. The skewness and kurtosis represent the degree of symmetry and distribution around the means; either the peakedness or flatness and distribution of data points in comparison to the normal distribution of the refined dataset [15,16]. The data presented in Table 1 failed to achieve homogeneity of variance and none of the data transformation approaches were employed because the mean effect size and weighting approaches described under Section 2.2 are robust against the violation of this assumption [17]. It is worth mentioning that some studies did not report all the variables of interest for the present study, making the number of observations on the variables presented to be inconsistent (Table 1). Some of the dietary rumen modulating strategies evaluated in the current study either reduced enteric methane (CH_4_) emissions while maintaining or improving productivity of ruminant livestock as reported in Table 2 and Table 3. The mean effect size (%) of evaluated dietary rumen modulating strategies on enteric CH_4_ production, CH_4_ yield and CH_4_ emission intensity, and production performance parameters (average daily gain, milk yield and milk quality) in ruminant livestock is presented in Table 4 and Table 5, respectively, to facilitate the interpretation of results. The information on studies used in the current analysis is reported in Appendix A. Tannins reduced (*p* < 0.05) total tract digestibility (−12%) and CH_4_ production (g/day) and CH_4_ yield (g/kg DMI) by 37% and 40%, respectively. Concentrate feeding improved (*p* < 0.05) dry matter intake (kg/day) and milk yield (g/kg DMI) on average by 23.41% and 20%, respectively, in ruminant livestock. On average, nitrate, saponin, oils, ether extract and biochar had no effect (*p* > 0.05) on dry matter intake (DMI), milk yield (MY), and average daily gain (ADG: beef cattle, buffaloes, deer, sheep, and goats). Equally, nitrate and ether extract had no effect (*p* > 0.05) on CH_4liveweight_ (g/kg liveweight gain) and CH_4_ production. Seaweed induced a decrease in ADG (*p* < 0.05) by 3.75%. Methane yield (g/kg DMI) was reduced (*p* < 0.05) in response to nitrate (−10.11%), tannin (−37%), oils (−7.12-%), seaweed (−35.34%), and 3-nitroxypropanol (3-NOP: −27.36%). Equally, methane production (g/day) was reduced (*p* < 0.05) in response to the use of saponin (−37.27%), tannins (−40%), biochar (−5.45%), and seaweed (−21.80%) in ruminant livestock. Nitrate, oils and 3-NOP reduced CH_4milk_ (g/kg milk) by 16.63%, 38.96% and 30.46%, respectively. Rumen fermentation parameters (i.e., propionate, butyrate, and A:P ratio) were influenced by the presence of tannins, oils and 3-NOP in diets of ruminant livestock. Tannins increased ruminal propionate (+10.90%) and butyrate (+9.80%) while reducing the A:P ratio (−5.70%). Similarly, 3-NOP increased propionate (+13.10%) and butyrate (+17.26%) with a reduction in the A:P ratio (−20.26%). In contrast, oils increased propionate (+2%) and reduced butyrate (−2.9%) and the A:P ratio (−3.80%) in rumen fermentation parameters. The production of milk fat (g/kg DMI) was increased in response to 3-NOP by 15%, whereas oils improved the yield of milk fat and protein (kg/d) by 16% and 20%, respectively.

## 4. Discussion

The enormous research interest in adopting a variety of strategies aimed to curb enteric CH_4_ emissions to reduce the environmental impact of ruminant livestock has yielded dynamic yet attainable results. However, these strategies have been reported to be effective in most in vitro trials and when verified in vivo, few have proved to impact CH_4_ emission. Also, the vast array of strategies awarding success of mitigating enteric CH_4_ emissions in ruminants are allied with detrimental impacts on the productivity of animals [4]. Thus, it is of prime importance to collectively examine the existing strategies with the aim to better understand their mode of action and facilitate the creation or meticulous attainment of feasible solutions towards circumventing CH_4_ emission while improving productivity and feed efficiency in ruminants. The current analysis identified strategies with the mode of action associated with methane formation-related pathways to single out mechanisms to explain the percentage mean effect size of different strategies on CH_4_ emission, rather than utilising a multi-faceted approach of potential drivers to discuss the results. This is due to their mode of action being similar across different types of ruminant species [9], facilitating the discussion of current findings using quantitative comparative examination.

### 4.1. Nitrate

Nitrate supply to ruminants in a meta-analysis study by Almedia et al. [18] revealed a 15% reduction in methane yield (g/kg DMI) without impairing total tract digestibility. Congruently, our findings revealed that nitrate reduced methane yield by 10.11% with no adverse effect on dry matter intake and total tract digestibility in ruminants. The reduction in CH_4_ emission intensity (g/kg milk) was 16% in our meta-analysis which is comparable to the study by Almedia et al. [18], who reported a range of reduction from 10.7% to 18.7%. The mechanism by which nitrate may reduce enteric CH_4_ emission is via outcompeting methanogenesis pathways for metabolic hydrogen (H_2_) resulting from the enteric microbial fermentation. This is due to the high affinity of nitrate with H_2_ as compared to carbon dioxide in the rumen [19]. Nitrate does not adversely affect dry matter intake and total tract digestibility, while it provides (to a minor extent) non-protein nitrogen (N) to the microbiota in the rumen [20]. Also, in developing countries, livestock are mostly maintained in roughage-based diets with little or no dietary non-protein nitrogen supplementation [21]. Therefore, nitrate supply can be a beneficial strategy to reduce CH_4_ emissions and provide a non-protein nitrogen source in livestock systems that rely on pastures that are inherently low in nitrogen content, especially in the tropics during the dry season.

### 4.2. Saponin

Although there were no effects of saponins on production performance parameters and other CH_4_ metrics (i.e., CH_4_ yield and CH_4_ per liveweight gain), our results revealed a 37% reduction in CH_4_ production due to saponins in ruminant livestock. In agreement with our results, a previous study by Abarghuei et al. [22] reported a similar reduction (37%) in CH_4_ production. Also, when *Samane saman* (containing saponins) was supplemented to steers fed a basal diet of rice treated with urea, there was a reduction in CH_4_ production of 50% [23]. However, *Samane saman* also contains tannins, therefore, its CH_4_ mitigating effect could be attributed to some unknown extent to other components rather than solely saponins. The mechanism of action in saponins to reduce CH_4_ production is through the detergent-like action interfering with the integrity of methanogenic archaea cell membranes [24]. In this view, the population and ability of methanogenic archaea to produce CH_4_ may be compromised and enteric fermentation pathways altered by redirecting more hydrogen towards alternative sinks. The literature reporting the impact of saponin associated protozoa influencing methanogens is intriguing, yet limited studies hinder a conclusive understanding of the phenomenon.

### 4.3. Tannin

The study conducted by Goel and Makkar [21] compared the effectiveness of tannins and saponins on reducing CH_4_ production. Their findings indicated that tannins were less effective than saponins, which is contrary to the results obtained in our study (−40% vs. −37%). However, our analysis demonstrated that tannins highly (>30%) reduce both CH_4_ production and CH_4_ yield by 40% and 37%, respectively, despite the 12% decrease in total tract digestibility. Similarly, Arndt et al. [11] reported a 12% decrease in both CH_4_ production and total tract digestibility. Goel and Makkar [21] noted that although tannins do reduce CH_4_ production in vivo, there is a drawback in terms of impaired animal productivity in view of the reduction in total tract digestibility. Additionally, in this study, fermentation parameters such as propionate and butyrate increased by 10.9% and 9.8%, respectively, while the acetate to propionate ratio decreased by 5.7%. The mechanism by which tannins affect methanogenesis can be partially explained by their direct toxic effect on methanogens and protozoa, as well as their ability to bind to polysaccharides, thereby adversely affecting fibre degradation in the rumen and increasing propionate production [25,26,27]. The reduction in the amount of dry matter degraded in the rumen leads to a decrease in the production of metabolic hydrogen, which is necessary for methanogenesis. Moreover, tannins directly reduce the population of methanogens through their toxic effect and by increasing rumen washout rates, resulting in a reduction of CH_4_ formation in the rumen [28]. To optimize feed utilization while using condensed tannins in ruminant diets, careful attention must be paid to protein concentration, ensuring it does not compromise real apparent total digestibility. Additionally, research suggests that the effectiveness of tannins varies with their origin, as some rumen microbes develop resistance mechanisms over time [29]. Tannins also form complexes with proteins under physiological ruminal pH conditions, which reduces the degradability of protein in the rumen, thereby improving bypass protein [21].

Although it is not evaluated in the current analysis, it is important to note that the shift of nitrogen excretion from urine to faeces attributed to tannin supply is significant and cannot be overlooked, despite the induced low dry matter digestibility. Therefore, it is necessary to evaluate the long-term effects of tannins on the overall productivity of ruminants [5]. This evaluation should be accompanied by determining the threshold dosage and source of tannins supplied in separate pulse doses, using a range of carriers in addition to the general approach of including it in a total mixed ration. These carriers should be tailored to the specific feeding systems prevalent in different regions; for instance, grazing is the most common system of rearing ruminants in sub-Saharan Africa [30]. Therefore, the carriers should be situation specific or should cater for conditions prevalent in a specific area, or, if possible, a range of areas.

### 4.4. Oils and Ether Extract

Both oils and ether extract contain polyunsaturated fatty acids that have been found to exhibit anti-methanogenic properties. Therefore, the combination of oils and ether extract facilitate the discussion of the results according to their mode of action in reducing CH_4_ emissions from ruminants. The findings of the current study revealed several interesting results regarding the effects of oils on ruminants and their milk production. The supply of oils to ruminants led to a reduction of 7.13% and 38.96% in CH_4_ yield and CH_4_ intensity (g/kg milk), respectively. Additionally, there was an increase of 16% and 20% in milk fat and protein production (kg) per day, respectively. These results are consistent with a study conducted by Madhavi et al. [31], which reported similar increases of 9.09% and 9.38% in milk fat and protein production, respectively, in response to a supply of oil in the diets of dairy cows. The reduction in CH_4_ intensity is due to an increase in the production of milk, thereby lowering the quantity of CH_4_ per unit of milk produced. This is attributed to the biohydrogenation process triggered by the rise in dietary energy from oils in the rumen, where hydrogens are captured and, in the case of dairy ruminants, utilised for the synthesis of milk fat. However, the mechanism behind the potential increase in milk protein remains not clearly known. Generally, it is believed that incorporating feedstuffs such as grains, fats, or oils in ruminant diets can stimulate milk production by providing high energy and redirecting additional energy from CH_4_ mitigation. The spared energy from inhibiting CH_4_ in livestock, even with a 25% reduction through feed additives, rarely yields significant improvements in production [32]. Theoretical calculations based on experimental data suggest a minimal and challenging-to-detect enhancement in net energy for production [32]. Despite the anticipated impact on animal energy balance, the observed gains in net energy are biologically insignificant at commonly reported inhibition rates. Methane reduction is linked to changes in organic matter digestibility and apparent digestible energy, complicating its relationship with energy metabolism. Studies reveal that targeting rumen methanogenesis does not consistently boost milk or growth productivity, with confounding factors like intake, digestibility, and rumen pH [33]. In essence, while there is theoretical premise, empirical evidence indicates limited and inconsistent effects on livestock production [34].

As expected, the findings of our study align with a meta-analysis conducted by Villar et al. [35] that examined effects of oil supplementation on dairy cows. The authors reported an 8.8% reduction in CH_4_ production, supporting the notion that oils can be an effective strategy for mitigating methane emissions. Benchaar et al. [36] reported a 25% reduction in the production of a gram of CH_4_ per kg of milk produced in lactating dairy cows, which is less than the reported 38.96% from our meta-analysis. However, Martin et al. [37] reported a slightly comparable 30% reduction in CH_4_ intensity in response to the oil supply in lactating dairy cows. The study of Beauchemin et al. [38] reported a similar observation of reduced CH_4_ intensity when cows were subjected to oils. The mechanism behind this reduction is believed to involve the disruption of methanogen cell membranes by the direct toxic effect of oils, leading to a decrease in methane production in the rumen. Briefly, polyunsaturated fatty acids in oils can undergo oxidation processes in the rumen. This oxidation can result in the generation of reactive oxygen species within the microbial cells causing oxidative stress, damaging cellular components and leading to cell death in ruminal microbes [39]. This disruption impacts their ability to carry out metabolic processes such as methane production.

On the other hand, our study did not find any significant impact of ether extract on both enteric methane production and animal performance parameters. This differs from the findings of a study by Giger-Reverdin et al. [40], which showed a reduction in CH_4_ yield in dairy cows fed diets with increased ether extract content. This reduction was attributed to the presence of unsaturated fatty acids (UFAs) in the ether extract, which promote the production of propionate and inhibit cellulolytic activity, thereby reducing methane production. The disparity between our results and those of Giger-Reverdin et al. [40] may be attributed to differences in the sources of ether extracts used in the respective studies, as well as variations in the average ether extract content of the diets. Our study had a lower average ether extract content than the study by Giger-Reverdin et al. [40], which could have contributed to the observed differences.

### 4.5. Concentrate Feeding

Dietary manipulation through the inclusion of concentrate in ruminant rations reduces the ratio of structural carbohydrates to starch. This improves diet digestibility and helps to reduce the amount of feed required per unit of animal product [41]. Some authors [42,43,44] have postulated that including concentrate in ruminant diets effectively reduces methane emissions. However, in general, including concentrate in ruminant diets at levels below 60% of the total diet would have no significant impact on methane emissions while only modestly affecting ruminal processes (Hegarty, personal communication). Nevertheless, Hristov et al. [45] reported that the use of concentrate as dietary intervention has a low (≤10%) to medium (10–30%) long-term ability to mitigate enteric methane emissions in ruminants when included at levels beyond 50%.

In the current study, our results are consistent with a grazing trial conducted by Jiao et al. [46] where feeding concentrate as a supplement feed sustained methane emission parameters while improving the dry matter intake and milk yield in Jersey dairy cows. Our analysis revealed that the supply of concentrate improved dry matter intake (kg/day) and milk yield (g/kg DMI) by 23.41% and 19%, respectively, in ruminant livestock. The increase in milk yield (+19%) observed in response to concentrate feeding in ruminants in the current study was also evident in the study of Schilde et al. [47], where dairy cows fed a high (58.2%) versus low (46.7%) concentrate diet had a higher (+14.57%) milk yield. Similarly, Angeles-Hernandez et al. [8] reported an increased milk yield in sheep fed a high concentrate diet compared to a high forage-based diet. In contrast to the findings of Schilde et al. [47], the current study did not show an increase in milk protein content. In fact, the milk protein yield decreased by 16%. Concentrates are typically more energy-dense than forages, providing a greater amount of metabolisable energy per unit of feed. This additional energy can be utilised by dairy ruminants for milk synthesis, resulting in an increased milk yield. Concentrates typically have higher protein levels and are more palatable than forages, resulting in improved dry matter intake and milk production as seen in the current study.

However, it is important to note that the reduction in milk protein content by −16% needs to be considered. The decrease in milk protein concentration can be attributed to the dilution effect caused by the increased milk yield. As milk production increases, the total amount of milk protein synthesized by the animal may still be higher despite the reduced concentration. Therefore, although the milk protein content decreases, the overall milk protein yield might not be significantly affected. Supporting this, Huhtanen and Hetta [48] reported a positive relationship between the level of concentrate intake and milk yield in a meta-analysis study. These results suggest that including concentrate in ruminant diets is an effective strategy to improve dry matter intake and milk yield. While there are no reported studies on the deleterious environmental impacts of including concentrate in ruminant diets, inclusion levels of concentrate beyond a certain threshold are associated with acidosis [49]. Moreover, an increase in dry matter intake is implicated in increasing the passage rate and reducing ruminal degradability [50], which could potentially increase the fermentable organic matter content in faecal excreta and subsequently greenhouse emissions [51]. However, the agronomic traits of the basal diet available, the amount, and the chemical composition of concentrate diets consumed by ruminants should be considered as underlying factors affecting the degree of degradability, passage rate, and ultimately the content of faecal excreta which are not reported in the current analysis.

### 4.6. Seaweed

Extensive research has been conducted both in the laboratory [52,53,54] and on animals such as sheep [55], beef cattle [56], and dairy cattle [57] to investigate the efficacy of seaweed (i.e., Asparagopsis) in reducing enteric CH_4_. The outcomes have shown a variation in CH_4_ reduction, likely due to a variation in diet quality (e.g., fibre content) as reported by Lean et al. [58] and the level of bromoform, a compound derived from seaweed responsible for the reduction in CH_4_ emissions. In alignment with the outcomes of our research, which exhibited a 35.3% decline in CH_4_ yield (g/kg DMI) in ruminant livestock, a meta-analysis conducted by Machado et al. [52] examining the efficacy of mainly Asparagopsis algae-based diets in beef cattle also indicated a 37% decrease in CH_4_ yield. Nevertheless, trials conducted on dairy cows demonstrated inconsistent effectiveness of Asparagopsis. According to Roque et al. [54] and Stefenoni et al. [57], the reported efficacy in CH_4_ yield was 43% and 80%, respectively, when Asparagopsis was included at levels of 1.84% DM and 0.5% DM, correspondingly. Also, the sole study of Li et al. [59] conducted in sheep reported a range of 15% to 81% CH_4_ yield mitigating potential when the inclusion level of Asparagopsis varied from 1.0% to 5.7% on DM basis. The meta-analysis study by Almeida et al. [18] reported a comparative higher mean reduction of 49% in CH_4_ yield across ruminant species subjected to variable feeding levels and diet quality. Such results could be attributed to factors such as diet quality and the inclusion level of seaweed, to name but a few. Nevertheless, certain varieties of seaweeds possess the ability to produce and enclose halogenated methane analogues within specialized gland cells. Among these seaweeds, Asparagopsis has been identified as having bromoform as the primary compound that suppresses methane emissions. Bromoform (CHBr_3_) and other halogenated methane analogues, such as bromochloromethane (BCM), inhibit the process of methanogenesis by binding to and isolating the prosthetic group necessary for methyl coenzyme M reductase (MCR), which is responsible for the final step in methanogenesis. Bromoform is considered toxic, and exposure to high levels of bromoform can, as a residue in milk or meat products, cause adverse health effects [53]. These effects can include central nervous system depression, liver and kidney damage, respiratory effects, and gastrointestinal disturbances [53].

### 4.7. Nitroxypropanol

Our study findings align with previous research conducted by Melgar et al. [60] which reported a reduction of 27% in CH_4_ yield with the use of 3-nitrooxypropanol (3-NOP). Similarly, our study observed a slightly higher reduction of 27.4% in CH_4_ yield from ruminants upon incorporating 3-NOP. This reduction in CH_4_ yield was accompanied by an increase in propionate (+13.1%) and butyrate (+17.3%) production, as well as a decrease in the acetate-to-propionate ratio (−20.3%) within rumen fermentation parameters. A decrease in CH_4_ yield achieved using 3-NOP did not negatively impact dry matter intake or milk yield. Long-term investigation conducted by Melgar et al. [60] consistently demonstrated that incorporating 3-NOP into dairy cattle diets did not result in any adverse effects on DMI or lactation performance. Similarly, Kim et al. [61] specifically focused on beef cattle and found no significant effect on DMI when 3-NOP was included. Our results revealed a 30.5% reduction in CH_4_ intensity (g/kg milk). Similarly, Melgar et al. [60] reported a 27.4% decrease in CH_4_ intensity. Melgar et al. [60] reported an increase of 4.83% in milk fat yield when 3-NOP was included in the rations of lactating dairy cows. Equally, the inclusion of 3-NOP was found to increase milk fat concentration in a meta-analysis study by Jayanegara et al. [62]. Consistent with this finding, we observed a noteworthy 15% increase in milk fat yield in response to 3-NOP. To understand the mode of action behind the reduction of CH_4_ emissions in response to 3-NOP, it is important to consider the normal process of archaeal-associated methane formation in the rumen. This process involves the docking of methyl-coenzyme M reductase with methyl-coenzyme M, resulting in the production of enteric CH_4_ [63]. In comparison to methyl-coenzyme M, 3-NOP exhibits a strong affinity for methyl-coenzyme M reductase and 3-NOP possesses a similar chemical structure to methyl-coenzyme M [38]. When administered to ruminants, 3-NOP replaces methyl-coenzyme M and prevents the formation of CH_4_ [38]. This occurs through the oxidation of the nickel in methyl-coenzyme M reductase by 3-NOP, preventing its binding to methyl-coenzyme M [64]. During this reaction, 3-NOP undergoes reduction in intermediate steps, including the formation of nitrate, nitrite, ultimately resulting in the production of 1,3-propanediol as 3-NOP is degraded in the rumen [64]. Our study findings, along with those of Melgar et al. [60] and Jayanegara et al. [6], highlight the significant reduction in CH_4_ intensity achieved through the utilization of 3-NOP in ruminant diets. This reduction is accompanied by favourable changes in rumen fermentation parameters, including increased propionate and butyrate production, and modified VFA composition. The redirection of energy towards milk fat synthesis contributes to the notable increase in milk fat yield. The consistent findings across these studies emphasize the potential of 3-NOP as a valuable tool in mitigating methane emissions from ruminants while maintaining or even enhancing animal productivity. The consistent administration of 3-NOP through its incorporation into total mixed diets fed to ruminants in confinement maximizes efficacy, while its use in grazing conditions is not yet effective. Furthermore, the mode of action of 3-NOP, involving its affinity for methyl-coenzyme M reductase and the subsequent prevention of CH_4_ formation, provides mechanistic insights into its effectiveness.

### 4.8. Biochar

Biochar is a charcoal-like product of the anaerobic combustion of vegetation under an extreme heat (pyrolysis) of between 350 °C to 1100 °C [65]. The porous nature and large Brunauer–Emmett–Teller (BET; 2–40 m^2^/g) surface area of biochar provides a site for ruminal microbiota allied with methanogenic–methanotrophic interaction, likely mediating CH_4_ oxidation [66]. The BET is the measure of the ability of a material to absorb gases [67]. The ability of biochar to trap gases in soils [68] could be inferred to the trapping of CH_4_ in the rumen as made evident in a study by Leng et al. [66], who reported a 22% reduction of CH_4_ production in “Yellow” cattle fed 0.6% of dietary biochar on a dry matter basis. Our findings demonstrate a 5.5% reduction in CH_4_ production (g/day) in ruminants which is in agreement with the findings of Leng et al. [66] who observed a similar reduction pattern but lower in magnitude. Conversely, Conlin [68] reported no change in CH_4_ production and production performance parameters of grazing beef cattle in response to an addition of 1–3% DM of biochar in their diet. Similarly, Winders et al. reported no effects of biochar on CH_4_ production and CH_4_ yield in beef steers. Likewise, supplementation of biochar in the diets of lambs had no effect on ADG or gain to feed ratio. Thus, due to the sporadic methane mitigation effect of biochars in the rumen, it is difficult to ascribe the specific mode of action of biochar associated with enteric CH_4_ mitigation in ruminants [69]. Regardless of this, it is postulated that if CH_4_ mitigation occurs in the rumen due to the inclusion of biochar in ruminant diets, the reduction could be attributed to the reduction in the rumen methanogenesis-associated protozoa population and/or role of biochar acting as an electron (e^−^) shuttle responsible for moving e^−^ between ruminal microbes or chemical acceptors and ruminal microbes [63]. In this study, the exact mechanism associated with the observed reduction in CH_4_ production due to biochar might likely be ascribed to one of the following two notions postulated by Leng et al. [66]. Firstly, it could be stated that biofilm associated with the surface area provided by the inert biochar may have supported the population density of anaerobic methanotrophs sufficiently for CH_4_ oxidation. Secondly, it is possible that biochar provided a site for the improved efficiency of microbial cell production through the closer association of microbial communities, thereby improving adenosine triphosphate (ATP) production and utilization [66]. However, our findings do not corroborate the second notion suggested by Leng et al. [66] as the efficiency of ATP utilization was not reflected in the improved production performance parameters (i.e., ADG and milk production) of ruminant livestock. Therefore, in this study, it is inferred that the observed reduction in CH_4_ production due to the use of biochar can be partly explained by the first mode of action described by Leng et al. [66].

## 5. Conclusions

This study revealed that the various dietary rumen manipulating strategies found to be effective in reducing enteric methane emission differed significantly in terms of the magnitude of emission reduction, mode of emission reduction and subsequent effect on production parameters. Among the identified effective CH_4_ mitigating strategies that do not adversely affect the production performance of ruminants, saponins have a high effect (>30%) on the reduction of CH_4_ production, while oil inclusion revealed a high effect in reducing the intensity of methane emission in dairy cows (lower CH_4milk_); nitrate and 3-NOP showed a medium effect (10–30%) in reducing the intensity of methane emission in dairy ruminants (CH_4milk_). Similarly, nitrate and 3-NOP has a medium effect in reducing methane yield while oil has a low effect (<10%) in reducing methane yield (CH_4yield_). On the other hand, concentrate feeding has revealed a beneficial effect by improving the production performance (DMI, MY and MPp) of ruminants without any significant effect on CH_4_ metrics in ruminant livestock. The inclusion of oil and 3-NOP has provided a co-benefit by improving MFy and MPy in dairy ruminants. Similarly, inclusion of 3-NOP provided co-benefit by improving MFp in dairy ruminants. The observed variation in the magnitude of CH_4_ reduction and mode of reduction call for further research on the combined use of two or more of the effective CH_4_ mitigating strategies in conjunction with evaluating their impact at farm-scale levels and assessing the sustainability of their long term use by quantifying net greenhouse gas emissions, through modelling or life cycle analysis, to mediate the adoption rate. Hence, it is advisable to adopt and upscale these strategies with caution, depending on the specific goal to be achieved in ruminant livestock systems.

## Figures and Tables

**Table 1:** Summary statistics of the refined complete dataset on dietary rumen modulating strategies, initial body weight, dietary composition, intake, total tract digestibility, average daily gain, milk parameters, feed conversion ratio, methane and fermentation parameters of ruminant livestock.

Parameter	*n*	Mean	Minimum	Maximum	Standard Error	CV	Skewness	Kurtosis
**Dietary rumen modulating strategies (g/kg)**								
Nitrate	39	19.5	0.30	223	5.59	179.48	5.46	32.28
Saponin	49	21	3.30	170	5.10	169.64	3.48	12.06
Tannins	112	54.3	0.10	272	6.15	119.95	1.24	1.03
Oils	104	20.3	0.05	50	1.49	74.59	0.15	−0.90
Ether extract	24	36.3	4.82	174	1.17	54.95	1.56	6.88
Concentrate feeding	28	467.3	33	800	36.74	41.61	−2.00	−0.46
Biochar	18	181	50	460	26.40	61.71	0.92	0.52
Seaweed	14	73	0.50	300	16.70	97	2.04	5.42
3-Nitroxypropanol	21	98.5	2.01	200	18.90	71.79	0.57	−1.26
**Initial body weight (kg)**	521	202	11.20	1405	10.32	116.83	1.29	0.67
**Dietary chemical composition (g/kg)**								
DM	231	766.3	173	956	13.44	26.65	−1.21	0.27
OM	292	919.6	753	970	1.86	3.45	−2.17	8.86
CP	473	157.6	27	850	2.69	37.07	4.26	41.97
NDF	438	414	86	781	6.49	32.80	0.50	−0.17
ADF	427	261.2	15.97	644	5.82	46.06	0.82	0.98
**Intake (kg/d)**								
DM	531	6.6	0.418	28.60	0.33	113.66	1.18	0.076
OM	276	0.9	0.435	18.20	0.09	165.11	8.83	87.02
CP	427	1.2	0.026	5.16	0.07	118.41	1.17	0.04
NDF	402	2.7	0.154	12.50	0.15	109.57	1.25	0.75
ADF	385	1.6	0.019	8.64	0.09	115.61	1.55	1.98
**Total tract digestibility (g/kg)**								
DM	324	632	410	866	4.23	12.05	0.04	0.06
CP	226	611	203	901	10.23	25.16	−0.98	1.10
NDF	355	509	211	854	6.11	22.61	−0.17	0.413
ADF	266	466	126	639	5.65	19.76	−0.41	0.64
EE	140	682	302	970	14	24.30	−0.45	−0.37
**ADG (g/day)**	254	172	−32	1600	14.14	130.94	3.06	11.44
**Milk production (kg/day)**	135	14.5	1.15	45.20	1.10	69.34	0.18	−0.94
**Feed conversion ratio (g/kg DMI)**	125	3.4	−15.77	145.72	1.24	404	8.85	90.53
**Milk yield (g/kg DMI)**	26	10.4	0.66	27.13	1.965	96.43	0.57	−1.38
**Milk quality**								
MFp (g/kg)	124	3.67	0.30	7.76	6.20	53.42	3.75	1.87
MFy (kg/d)	103	3.2	0.001	7.33	0.23	7.69	−1.5	−0.5
MPp (g/kg)	91	0.5	0.01	8.0	0.026	4.44	−0.16	−1.36
MPy (kg/d)	124	3.7	0.30	7.76	0.18	6.85	1.29	1.13
**Methane**								
g/kg LWG	40	117.7	1.52	482	18.54	99.66	1.15	0.97
g/day	252	74.7	2.81	635	8.29	176.21	2.59	6.04
g/kg DMI	220	22.4	4.70	58.80	0.503	33.33	1.10	4.35
g/kg milk	35	12.3	0.33	60.41	29.3	145.77	1.58	1.52
**Fermentation parameters**								
Acetate	345	41.5	7.10	94.48	1.11	49.71	0.383	−1.07
Butyrate	345	13	1.35	35.20	0.39	54.96	0.85	−0.11
Propionate	345	7.6	1.12	30.71	0.266	64.90	1.78	4.52
Acetate: propionate ratio	321	3.5	0.99	7.32	0.066	33.63	0.86	0.79
pH	140	6.5	5.9	7.11	0.02	4.32	−0.026	−1.06

*n*, number of observations; CV, coefficient of variation; DM, dry matter; CP, crude protein; NDF, neutral detergent fibre; ADF, acid detergent fibre; EE, ether extract; ADG, average daily gain (data exclusive of dairy ruminants); LWG, liveweight gain (data exclusive of dairy ruminants); DMI, dry matter intake; MFp, production of milk fat; MFy, yield of milk fat; MPp, production of milk protein; MPy, yield of milk protein. Skewness and kurtosis represent degree of asymmetry and distribution around their means, respectively. For each skewness and kurtosis value of ±2 × SE suggests normal distribution of data.

**Table 2:** Effect of dietary rumen modulating strategies on enteric methane and rumen fermentation parameters from ruminant livestock.

Dietary Rumen Modulating Strategies	Enteric Methane					Fermentation Parameters					Ruminant Type	Feeding System
	CH_4liveweight_	CH_4milk_	CH_4yield_	CH_4production_		Acetate	Propionate	Butyrate	Acetate/Propionate Ratio	pH		
**Nitrate**	NE	−16.63%	−10.11%	NE		ND	ND	ND	ND	ND	Beef, dairy, goats, and sheep	Confined and grazing
**Saponin**	NE	ND	NE	−37.27%		ND	ND	ND	ND	ND	Beef, buffaloes, goats and sheep	Confined and grazing
**Tannin**	NE	ND	−37%	−40%		NE	+10.90%	+9.80%	−5.70%	NE	Beef, buffaloes, deer, goats and sheep	Confined and grazing
**Oils**	ND	−38.96	−7.13%	NE		NE	+2%	−2.90%	−3.80%	NE	Beef, buffaloes, deer, goats and sheep	Confined and grazing
**Ether extract**	NE	NE	ND	NE		NE	NE	NE	NE	NE	Dairy, deer and goats	Confined
**Concentrate feeding**	ND	NE	NE	NE		NE	NE	NE	NE	NE	NE	Confined and grazing
**Biochar**	ND	ND	NE	−5.45%		NE	NE	NE	NE	NE	Beef, goats and sheep	Confined
**Seaweed**	ND	ND	NE	−35.34%		−21.80%	ND	ND	ND	ND	Beef, goats and sheep	Confined
**3-Nitroxy propanol**	ND	−30.46	−27.36%	NE		NE	+13.10	+17.26%	−20.26%	NE	Dairy, deer, and goats	Confined

CH_4liveweight_, gram of methane produced per kilogram (kg) of liveweight gain; CH_4milk_, gram of methane produced per kg of milk produced; CH_4yield_, gram of methane produced per kg of dry matter intake (DMI); CH_4production_; gram of methane produced per day; ND, no data; NE, no effect; positive values imply an increase in response to dietary rumen modulating strategies while negative values imply a reduction on enteric methane and rumen fermentation parameters from ruminant livestock at *p* < 0.05.

**Table 3:** Effect of dietary rumen modulating strategies on production performance and milk quality parameters of ruminant livestock.

Variables	Production Performance							Milk Quality				Ruminant Livestock	Feeding System
	DMI	TTDIG	ADG	MP	FCR	MY		MFp	MFy	MPp	MPy		
**Nitrate**	NE	NE	NE	NE	NE	NE		NE	NE	NE	NE	Beef, dairy, goats, and sheep	Confined and Grazing,
**Saponin**	NE	NE	NE	NE	NE	NE		ND	ND	ND	ND	Beef, buffaloes, goats, and sheep	Confined and grazing
**Tannin**	NE	−12	NE	NE	NE	NE		NE	NE	NE	NE	Beef, buffaloes, dairy, goats, and sheep	Confined and grazing
**Oils**	NE	NE	NE	ND	NE	NE		NE	+16	NE	+20	Beef, buffaloes, dairy, goats, and sheep	Confined
**Ether extract**	NE	ND	NE	ND	NE	ND		NE	NE	NE	NE	Beef, buffaloes, dairy, goats, and sheep	Confined
**Concentrate feeding**	+23.41%	ND	NE	NE	NE	+19%		NE	NE	+16.25%	NE	Dairy, deer, and goats	Confined and grazing
**Biochar**	NE	NE	NE	ND	NE	NE		ND	ND	ND	ND	Beef, goats and sheep	Confined
**Seaweed**	NE	NE	−3.75	ND	ND	NE		NE	NE	NE	NE	Beef and dairy	Confined
**3-Nitroxy propanol**	NE	NE	NE	ND	NE	NE		+15	NE	NE	NE	Beef and dairy	Grazing and confined

DIM, dry matter intake (kg/d); TTDIG, total tract digestibility (g/kg); MP, milk production (kg/d); ADG, average daily gain (g/day: data exclusive of dairy ruminants); FRC, feed conversion ratio (ADG/kg DMI: data exclusive of dairy ruminants); MY, milk yield (g of milk/kg DMI); MFp, production of milk fat (kg/d); MFy, yield of milk fat (g/kg DMI); MPp, production of milk protein (kg/d); MPy, yield of milk protein (g/kg DMI); ND, no data; NE, no effect (*p* > 0.05); positive values imply an increase in response to rumen modulating strategy compared to the control group while negative values imply a reduction on production performance and milk quality parameters of ruminant livestock at *p* < 0.05.

**Table 4:** Mean effect size (%) of potential dietary rumen modulating strategies on enteric methane emission and rumen fermentation parameters in ruminant livestock.

Parameters	Methane Emission								Rumen Fermentation Parameters									
	CH_4_ g/kg LWG		CH_4_ g/kg DMI		CH_4_ g/d		CH_4_ g/kg Milk		Acetate		Propionate		Butyrate		A/P Ratio		pH	
Dietary Rumen Modulating Strategies	%	*p*-Value	%	*p*-Value	%	*p*-Value	%	*p*-Value	%	*p*-Value	%	*p*-Value	%	*p*-Value	%	*p*-Value	%	*p*-Value
**Nitrate**	−28	0.169	−10	0.007	1.80	0.91	−16.63	0.10	ND	ND	ND	ND	ND	ND	ND	ND	−17	0.1
**Saponin**	−2.52	0.87	−4.29	0.32	−37	0.04	ND	ND	ND	ND	ND	ND	ND	ND	ND	ND	ND	ND
**Tannin**	−6.30	0.77	−37	<0.001	−40	0.003	ND	ND	−6.1	0.34	10.91	<0.001	−9.83	0.07	−5.66	0.01	ND	ND
**Oils**	ND	ND	−7.13	0.003	−28	0.12	−38	0.01	−0.4	0.96	2	0.02	−2.92	0.07	−3.81	0.04	−39	0.01
**Ether extract**	−17	0.28	ND	ND	−0.07	0.99	−15.66	0.09	0.17	1.0	0.66	0.64	−0.19	0.94	4.68	0.15	−15.6	0.09
**Concentrate**	ND	ND	−3.96	0.32	7.07	0.45	−11	0.27	−1.7	0.94	3.98	0.25	1.69	0.78	−3.41	0.74	−11	0.27
**Biochar**	ND	ND	−4.24	0.14	−5.45	0.05	ND	ND	1.03	0.94	2	0.35	1.94	0.87	1.58	0.59	ND	ND
**Seaweed**	ND	ND	−35	0.005	−22	0.08	ND	ND	ND	ND	ND	ND	ND	ND	ND	ND	ND	ND
**3-Nitroxy propanol**	−4.8	0.79	−27	0.002	ND	ND	−30	0.001	−8.5	0.64	+13.1	<0.001	17.26	<0.001	−20	0.001	−30	0.18

CH_4_ g/kg LWG, methane per liveweight gain; CH_4_ g/kg DMI, methane yield; CH_4_ g/d, methane production; CH_4milk_ g/kg milk, methane production per kilogram of milk produced; A/P, acetate to propionate ratio; ND, no data; positive values imply an increase in response to rumen modulating strategy compared to the respective control while negative values imply a reduction at *p* < 0.05.

**Table 5:** Mean effect size (%) of potential dietary rumen modulating strategies on production performance parameters in ruminant livestock systems.

Parameters	DMI		TTDIG		MP		FCR		MY		ADG		MF g/kg		MF kg/d		MP g/kg		MP kg/d	
Dietary Rumen Modulating Strategies	%	*p*-Value	%	*p*-Value	%	*p*-Value	%	*p*-Value	%	*p*-Value	%	*p*-Value	%	*p*-Value	%	*p*-Value	%	*p*-Value	%	*p*-Value
**Nitrate**	11.70	0.20	−0.16	0.97	1.45	0.78	−7.01	0.77	2.74	0.85	−8.24	0.94	2.74	0.70	2.74	0.75	2.74	0.44	2.74	0.86
**Saponin**	−0.10	1.00	−0.11	0.96	−7.23	0.81	2.25	0.86	ND	ND	−16	0.86	ND	ND	ND	ND	ND	ND	ND	ND
**Tannin**	−1.07	0.99	−12	0.01	−0.03	1.00	−7.87	0.44	3.84	0.77	0.88	0.99	6	0.36	2.51	0.88	−0.61	0.72	ND	ND
**Oils**	1.42	1.00	−2.25	0.20	ND	ND	−8.21	0.31	6.57	0.17	69.34	0.26	0.17	0.98	16.3	<0.001	ND	ND	20	0.008
**Ether extract**	7.45	0.25	ND	ND	ND	ND	−0.71	0.94	ND	ND	0.02	1.00	−8	0.14	1.46	0.77	−0.45	0.83	−3	0.73
**Concentrate**	+23.41	0.10	ND	ND	2.68	0.68	11.83	0.45	+19.90	<0.001	60.58	0.43	−3	0.66	3.65	0.77	−16	0.01	9.28	0.58
**Biochar**	−3.72	0.20	0.380	0.88	ND	ND	−3.72	0.83	18.41	0.21	ND	ND	ND	ND	ND	ND	ND	ND	ND	ND
**Seaweed**	−2.16	0.23	1.38	0.33	ND	ND	ND	ND	6.90	0.30	−3.75	0.004	0.14	0.98	0.65	0.82	0.69	0.86	ND	ND
**3-Nitroxy propanol**	−0.21	0.97	1.80	0.87	ND	ND	2.64	0.9	−0.94	0.87	−7.42	0.85	1.17	0.86	15	0.007	−0.16	0.97	0.64	0.97

DMI, dry matter intake (kg/d); TTDDIG, total tract digestibility (g/kg); ADG, average daily gain (g/day: data exclusive of dairy ruminants); FRC, feed conversion ratio (ADG/kg DMI; data exclusive of ruminants); MY, milk yield (g of milk/kg DMI); MFp, production of milk fat (kg/d); MFy, yield of milk fat (g/kg DMI); MPp, production of milk protein (kg/d); MPp, yield of milk protein (g/kg DMI); ND, no data; positive values imply an increase in response to rumen modulating strategy compared to the respective control while negative values imply a reduction at *p* < 0.05.

## Data Availability

Data is unavailable due to privacy.

## Appendix A

**Table A1:** Studies included in the meta-analysis.

Study No.	Reference	Animal Species	Adaptation Period (days)	Experimental Design
1	Terry et al. [70]	Beef heifers	14	Latin square
2	Doreau et al. [71]	Beef bulls	14	RBD
3	Mirheidari et al. [72]	-She-ep	14	CRD
4	Leng et al. [67]	Beef heifers	21	Factorial
5	Lunsin et al. [73]	Dairy cows	7	Latin square
6	Singla et al. [74]	Buffaloes	10	CRD
7	Marino et al. [75]	Sheep	n.r	Factorial
8	Kim and Kim. [76]	Beef	n.r	Factorial
9	Ortiz-Chura et al. [77]	Dairy calves	30	Crossover
10	Al-kindi et al. [78]	Goats	21	CRD
11	Winders et al. [79]	Beef	8	CRD
12	Van Wyngaard et al. [44]	Dairy cows	14	Factorial
13	Valenti et al. [80]	Sheep	n.r	CRD
14	McAvoy et al. [81]	Sheep	8	CRD
15	Liu et al. [82]	Sheep	14	Factorial
16	Van et al. [83]	Goats	14	Latin square
17	Pen et al. [84]	Sheep	14	Latin square
18	Lind et al. [85]	Sheep	14	CRD
19	Wang et al. [86]	Sheep	8	CRD
20	Mirheidari et al. [74]	Sheep	14	CRD
21	Animut et al. [87]	Goats	28	CRD
22	Conlin. [69]	Beef	14	Latin square
23	Liu et al. [88]	Sheep	n.r	CRD
24	Poteko et al. [89]	Dairy cows	21	CRD
25	Mirzaei-Alamouti et al. [90]	Dairy cows	14	CRD
26	El-Essawy et al. [91]	Goats	15	Latin square
27	Gerlach et al. [92]	Sheep	14	CRD
28	Jimenez et al. [93]	Goats	10	CRD
29	Molina-Botero et al. [94]	Dairy heifers	13	Latin square
30	Adejoro et al. [95]	Sheep	21	Factorial
31	Nasehi et al. [96]	Sheep	14	CRD
32	Puchala et al. [97]	Goats	14	CRD
33	Zhang et al. [98]	Sheep	14	CRD
34	Vázquez-Carrillo et al. [99]	Beef	28	Latin square
35	Thirumalaisay et al. [100]	Sheep	37	CRD
36	Barbosa et al. [101]	Goats	15	CRD
37	Heidarian et al. [102]	Goats	30	CRBD
38	Hulsof et al. [103]	Beef	16	Crossover
39	Zhang et al. [104]	Sheep	14	CRD
40	Van Zijderveld et al. [105]	Dairy cows	28	Crossover
41	Yang et al. [106]	Goats	15	Latin square
42	Granja-Salcedo et al. [107]	Beef	n.r	Crossover
43	Hollman et al. [108]	Dairy	21	Latin square
44	Hundal et al. [109]	Goats	n.r	Crossover
45	Paengkoum et al. [110]	Goats	14	Factorial
46	Nguye et al. [111]	Sheep	15	Crossover
47	Na et al. [112]	Goats and deer	7	Latin square
48	Animut et al. [89]	Goats	3	CRD
49	Pilajun and Wanap [113]	Buffaloes	7	CRD
50	Anassori et al. [54]	Sheep	10	Crossover
51	Benchaar et al. [36]	Dairy cows	18	Latin square
52	Guyader et al. [114]	Dairy cows	14	Crossover
53	Klevenhusen et al. [115]	Sheep	14	Latin square
54	Malik et al. [116]	Sheep	30	RBD
55	Pilajun and Wanap [117]	Buffaloes	7	Latin square
56	Verma et al. [118]	Buffaloes	n.r	Crossover
57	Tomkins et al. [119]	Beef steers	14	Latin square
58	Polyorach et al. [120]	Dairy heifers	14	Latin square
59	Castro-Montoya et al. [121]	Dairy cows	14	Latin square
60	Hundalet et al. [122]	Buffaloes	14	CRD
61	Inamdar et al. [123]	Buffaloes	n.r	CRD
62	Yatoo et al. [124]	Buffaloes	n.r	CRD
63	Yang et al. [125]	Dairy cows	11	Latin square
64	Meale et al. [126]	Dairy cows	11	Latin square
65	Manasri et al. [127]	Beef steers	n.r	Latin square
66	Manh et al. [128]	Dairy cows	n.r	Latin square
67	Chaves et al. [129]	Sheep	n.r	CRD
68	Beauchemin and McGinn [130]	Beef steers	n.r	Latin square
69	Matloup et al. [131]	Dairy cows	n.r	CRD
70	Wall et al. [132]	Dairy cows	7	CRD
71	Hristov et al. [51]	Dairy cows	14	Latin square
72	Benchaar et al. [36]	Dairy cows	14	Latin square
73	Giannenas et al. [133]	Dairy Sheep	7	CRD
74	Malik and Singhal [134]	Buffaloes	14	Crossover
75	Chiofalo et al. [135]	Sheep	n.r	CRD
76	Munoz et al. [136]	Dairy cows	21	Crossover
77	Olijhoek et al. [137]	Dairy cows	19	Crossover
78	Wang et al. [138]	Sheep	11	Crossover
79	Alves et al. [139]	Dairy cows	21	CRD
80	Tekippe et al. [140]	Dairy cows	21	Crossover
81	Sun et al. [141]	Beef steers	14	Crossover
82	Abdalla et al. [142]	Sheep lambs	n.r	CRD
83	Chilliard et al. [143]	Dairy cows	n.r	Latin square
84	Patra et al. [144]	Sheep	n.r	RBD
85	Ramirez-Restrepo et al. [145]	Beef steers	56	Crossover
86	Mao et al. [146]	Sheep	n.r	Factorial
87	Bayat et al. [147]	Dairy cows	14	Latin square
88	Aguerre et al. [148]	Goats	14	Latin square
89	Olijhoek et al. [149]	Dairy cows	19	Crossover
90	Jiao et al. [46]	Dairy cows	n.r	Crossover
91	Seremula. [150]	Sheep	14	Latin square
92	Haisan et al. [151]	Dairy cows	20	Latin square
93	Vyas et al. [152]	Beef heifers	13	Latin square
94	Romero-Perez et al. [153]	Beef heifers	14	Latin square
95	Alemu et al. [154]	Beef	16	Crossover
96	Zhang et al. [155]	Beef heifers	13	Latin square
97	Kinley et al. [56]	Beef steers	n.r	RIBD
98	Stefenoni et al. [57]	Dairy cows	21	Latin square
99	Singh et al. [156]	Dairy cows	56	CRD
100	Roque et al. [54]	Dairy cowa	n.r	Latin square
101	Barbosa et al. [103]	Goats	15	CRD
102	Phongphanith and Preston [157]	Beef cattle	15	Factorial
103	Terler et al. [158]	Dairy cows	n.r	Latin square

n.r, not reported; RBD, randomized block design; RIBD, randomized incomplete block design; CRD, complete randomized design.
